# Age of Acquisition Effect: Evidence From Single-Word Reading and Neural Networks

**DOI:** 10.32598/bcn.9.10.120

**Published:** 2019-03-01

**Authors:** Ahmad Sohrabi

**Affiliations:** 1. Department of Psychology, Faculty of Humanities and Social Sciences, University of Kurdistan, Sanandaj, Iran.

**Keywords:** Reading, Neural networks, Age of acquisition, Word recognition, Connectionism

## Abstract

**Introduction:**

Many studies show that words learned early in life are read more easily than the ones learned later and are less vulnerable to brain damage.

**Methods:**

the first part of the current study, 25 primary school students in the 5th grade read the word groups learned initially during a previous grade. The words used in the experiments were 327 Farsi monosyllable words matched on the other factors involved in Farsi word naming.

**Results:**

The analysis of covariance (the consistency and frequency as covariates) showed that words learned in earlier grades were read more easily than the ones learned later, showing the known effect of the Age of Acquisition (AoA). In the second part of the study, it was tried to simulate AoA in word naming by a neural network model developed earlier based on connectionist approach. While previous studies used random patterns, in the current study words from primary school books were used. Likewise, words learned early by the model were read better than words learned later. However, there was a failure in replicating previous simulation of AoA in English reading by an algorithm called Quick prop for Farsi. In addition, the model was lesioned by removing some hidden units to see its effect on word reading. As a result, words learned earlier were less vulnerable to damage compared with the ones learned later.

**Conclusion:**

The findings showed that words learned earlier, compared to those learned later, were read better and were less vulnerable to damage. These effects are explained by considering the nature of learning in neural networks trained by error back-propagation.

## Highlights

- Children read Farsi words learned in earlier grades better than the words learned later.
- The neural network model also showed a similar performance in reading Farsi words.
- The model with quick prop algorithm failed to show the same effect with Farsi words.
- In the lesioned model, the words learned earlier were less affected by the damage than the words learned later.
- These effects are explained by the nature of back-propagation and sigmoid function.

## Plain Language Summary

Both children and artificial computerized brains known as neural networks read words that appeared at early grades better than the words appeared at later grades. This reflects an age acquisition effect. However, when the model uses quick prop algorithm, it fails to show the same effect with Farsi words, unlike the previous studies results on English words. Moreover, when some neurons in the artificial model are destroyed, the words learned earlier are less affected by damage than the words learned later. These effects are explained by the mathematical nature of the learning in artificial brains.

## 1. Introduction

If the important variables in word recognition such as consistency, frequency, and word length are equal, it is shown that still the words learned early in life are read better than the ones learned later and are less vulnerable to brain damages. These effects are also shown in other cognitive processes including object naming, face recognition, and spoken word recognition (Morrison & Ellis, 1995; Gerhand & Barry, 1998; Zevin & Seidenberg, 2004; Izura et al., 2011; Wilson, Ellis, & Burani, 2012).

In several cognitive domains, early learning can result in a decrease in plasticity, which limits the ability to acquire new information. Phonological acquisition is a good example (Werker & Tees,1984), i.e. learning the phonological structure of a language lessens the ability to learn new phonetic contrasts (e.g. in a second language).

Likewise, studies show that the ability to learn the morphology and syntax of a language drops steadily after approximately seven years of age (Flege, Yeni-Komshian, & Liu, 1999). However, other faculties such as lexical acquisition do not seem to be equally age-dependent (Markson & Bloom, 1997; McCandliss, Posner, & Givon, 1997).

Carroll and White (1973) found a relationship between word learning age and later age processing speed. They showed that object naming latency had a high correlation with the age at which children learn the different object names. Through multiple regression analysis they further found that age of acquisition was the only significant independent variable to predict the naming latency.

The better performance in the case of words learned in early childhood is not due to more exposure (called frequency) in that time and later (called cumulative frequency), but has its own effect. Therefore, for example, words learned in early grades of primary school (e.g. grades 1 and 2) are recognized and read better than the words learned later (e.g. grades 4 and 5). There are other important factors involved in word recognition such as frequency, consistency (Coltheart’s N), and length of words (Coltheart, Davelaar, Jonasson, & Besner, 1997; Seidenberg, 1985; Cited in Sohrabi, 1999). The highly frequent and/or consistent words are better recognized than the low frequent and/or inconsistent ones.

Similar to many cognitive processes, word reading is simulated and explained well by connectionist model or artificial neural network (Seidenberg & McClelland, 1989; Zorzi, Houghton, & Butterworth, 1998; Sohrabi, 2001; Zevin & Seidenberg, 2004).Thus the current study aimed at investigating word naming in human subjects and simulating the connectionist models.

## 2. Methods

### 2.1. Investigating the age of acquisition effects by human data in primary school

This part of the study aimed at showing an effect of Age of Acquisition (AoA) controlling other important factors in naming words in primary school books in the 5^th^ grade students.

#### 2.1.1. Materials

The items were 327 words from primary school books. All words were grouped based on their initial appearance in one of the five grades. In addition, two other important factors were also considered: consistency by Coltheart N, frequency by objective measurement in primary school books as a good estimate of words that children of a small town in Iran are exposed to in primary school. All words were monosyllable.

#### 2.1.2. Participants

The study was conducted on 25 subjects of the fifth grade nearly at the end of the academic year. All participants were male and had no problems in speech and vision. At the end of the experiment, playing a computer game and an ice-cream were offered to all participants.

#### 2.1.3. Procedure

All words were printed in an MS-Windows font with size 100 points each in a 3”×6” card. The cards were put in random order. Subjects, sat in front of the experimenter, one by one, in a quiet room and all words were presented to them after the following instruction: “You will see some words from the primary school, each on a card. Read them aloud, as soon as possible, when they are shown one by one”. The experiment took about 25 minutes for each subject. The reading errors were recorded by an assistant on a three-level scale: Failure (3), failure with correction (2) and considerable delay (1).

### 2.2. Simulating AoA by a connectionist model

#### 2.2.1. Methods

Neurocomputational modeling was employed to simulate and explain cognitive processes (McClelland, McNaughton, & O'reilly, 1995; Seidenberg & McClelland, 1989; Zorzi et al., 1998; Zevin & Seidenberg, 2004; Sohrabi & West, 2009; Ludvig, Sutton & Kehoe, 2012) and lower and higher level neural functions (Daneshparvar & Daliri, 2012; Soltanzadeh & Daliri, 2014; Friston & Frith, 2015). However, the AoA was not modeled until recent years (Ellis & Lambon-Ralph, 2000). Previously, McClelland et al. (1995) simulated the graded improvement of children’s lexicon. They examined the outcomes of adding a novel concept (penguin) after training the network. This was done using either focused or interleaved learning. With focused learning, the system is presented to new knowledge without interleaving with old knowledge, i.e. no further exposure to the earlier training set. Under this condition, information about penguins was learned rapidly but at the cost of the pre-existing knowledge. In other words, the model underwent catastrophic interference. However, in the case of interleaved learning, i.e. the model being exposed to the old material alongside the new, the new information was learned without costing the old.

Ellis and Lambon-Ralph (2000), based on such findings, studied the AoA effect on random patterns through some simulations. They showed that AoA effect was different from cumulative frequency and attributed it to inevitable consequence of losing flexibility in artificial neural networks as it is the case for matured subjects. This is due to error back-propagation algorithm that changes the connections weights at the early training more than later training. Therefore, early training has more effects than late one. In this part of the study, using words from primary school books with their real frequency, some simulations were carried out to compare with those of human data.

#### 2.2.2. Architecture of the model

A distributed connectionist model based on Seidenberg and McClelland (1989) was used adopted for the Farsi language in a previous work (Sohrabi, 2001). In such models neuron-like units are used to connect input (letters) and output (phonemes). In the model, letters and phonemes are distributed among words, for each one a certain pattern of activation is involved. The input layer is 33 letters in the Farsi language with Arabic script and its output layer is 28 phonemes (in contrast to English, Farsi has less phonemes than letters). For better representation of rimes that have important roles in reading Farsi (Sohrabi, 1999), similar to that of English (Coltheart et al., 1997), four slots of units were considered for input and output layers similar to that of Zorzi (1998) and Sohrabi (2001).

**Figure 1.**
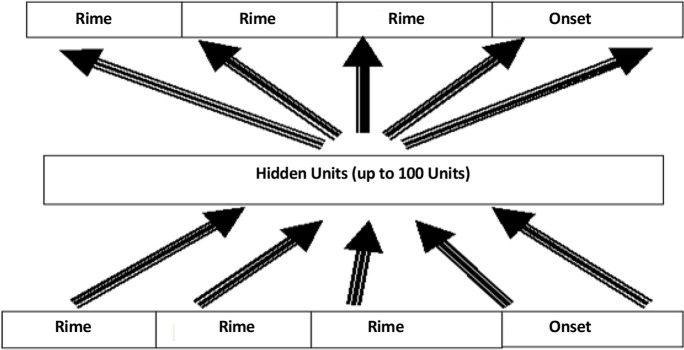
Distributed connectionist model The model based on Seidenberg and McClelland (1989) was used, which was adopted for the Farsi language in a previous work (Sohrabi, 2001).

Additionally, since Farsi has deep (quasi-regular) orthography (Sohrabi, 1999), a hidden layer including 100 units was used. Thus, a model with an input layer of 132 units and an output layer of 112 units was used in all simulations (Figure 1). At the start of the training, connections were weighted initially by random values between +0.5 and −0.5. Then the model was trained by error back-propagation algorithm (Rumelhart, Hinton, & Williams, 1986). The learning rate was 0.05 in all simulations except for 1, 2, and 3 in which it was 0.01. The momentum value that speeds up the learning was 0.9 in all simulations, but simulations 5, in which it was omitted to see its effect.

## 3. Results

### 3.1. Human data

Mean errors for each word made by all subjects were analyzed by analysis of covariance (item analysis) as a dependent factor. Five grades at which the words were initially learned made the five levels of fixed factor.

**Figure 2.**
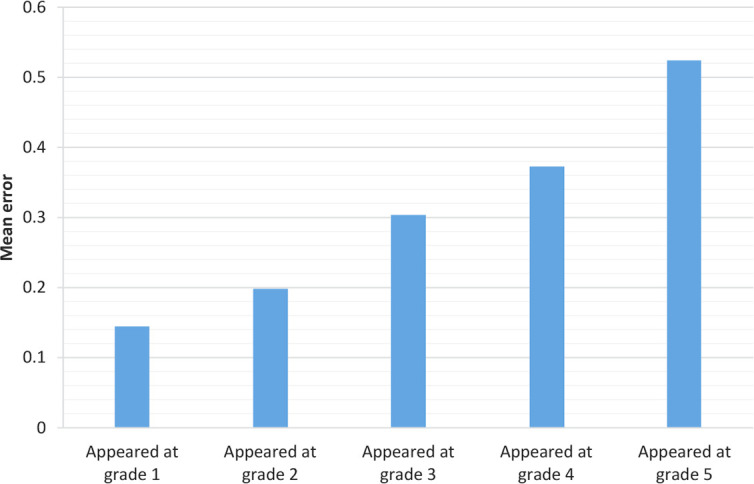
Mean error for word groups

Consistency and frequency were two covariates found as important factors in reading Farsi words by a multiple regression analysis (Sohrabi, 1999).The frequency, as a covariate, was not significant, but was included in the analyses to control variance; as shown in Tables 1 and 2 and Figure 2. The consistency covariate was significant; therefore, AoA was mainly due to inconsistent words.

**Table 1.** Mean and SD of reading errors in five word groups

**Word Group**	**N**	**Mean±SD**
Appeared at grade 5	78	0.524±0.5779
Appeared at grade 4	49	0.3724±0.5393
Appeared at grade 3	56	0.3036±0.4357
Appeared at grade 2	62	0.1986±0.2913
Appeared at grade 1	82	0.1441±0.3218
Total	327	0.3066±0.4649

**Table 2.** Analysis of covariance on reading errors in five word groups

**Source**	**SS**	**df**	**MS**	**F**	**Sig.**
Frequency	0.123	1	0.123	0.696	0.405
Consistency	7.089	1	7.089	40.166	0.000
AoA	5.476	4	1.369	7.757	0.000
Error	56.476	320	0.176		

As can be observed in Tables 1 and 2, word initially learned in early grades had less errors compared to the ones learned in later grades. The effect of word groups was significant (F_4,320_=7.757; MSE=0.17; P<0.001).

Though the differences between immediate grades were not significant (except for 4 and 5), other contrasts were significant. Additionally, there was a significant linear trend for AoA effect. Controlling other factors, the earlier the word, the less the error.

### 3.2. Modeling data

#### 3.2.1. Simulation 1

**Figure 3.**
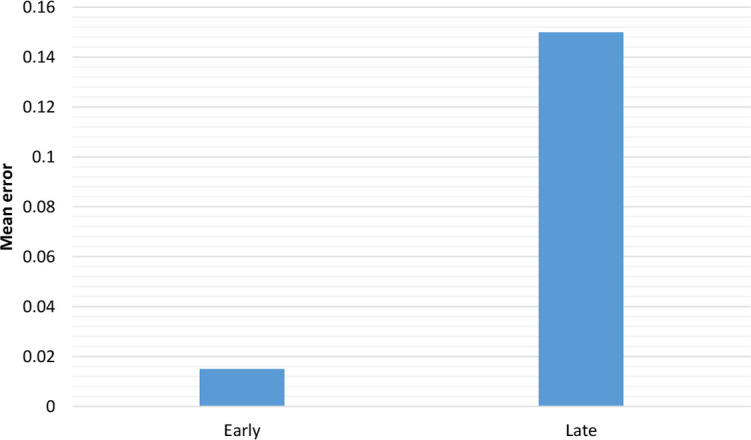
The errors for early and late after 300 epochs

This simulation was intended to show an effect of AoA even by controlling the frequency effect. First, the model was trained for 150 epochs on 82 early words and then for 150 epochs on 82 early words (from grade 1) as well as 87 late words (from grade 2). The late words were trained twice, to be equal to the early ones in frequency. The words were trained in a random order. The result showed that early words had significantly less errors than the late ones (Figure 3).

#### 3.2.2. Simulation 2

**Figure 4.**
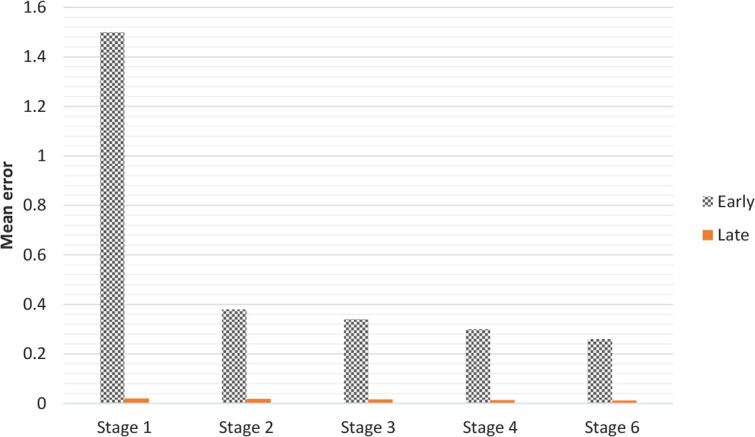
The errors for early and late after 600 epochs

This simulation was the same as simulation 1. But here, training the mixed words groups continued twice, as shown in Figure 4. The effect of AoA remained superior after several epochs as shown in human beings after some decades (Ellis & Morrison, 1998).

#### 3.2.3. Simulation 3

**Figure 5.**
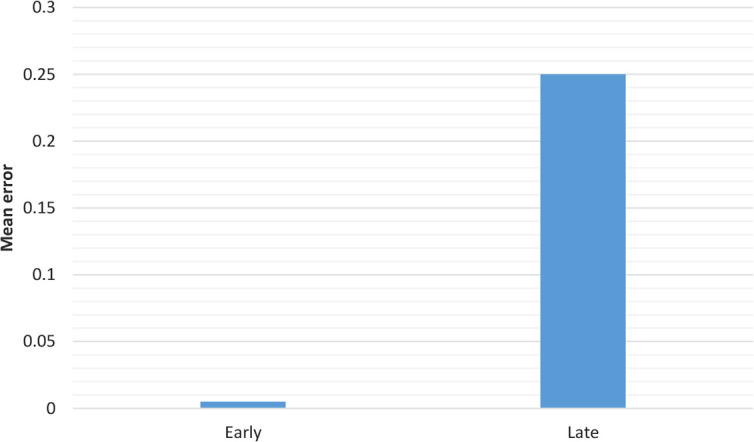
The errors for early and late after 300 epochs with reversed order

The aim of this simulation was to control the possible difference between the selected two groups. Thus, the order to train the two groups reversed. Again, the gain for the early ones was observed (Figure 5).

#### 3.2.4. Simulation 4

**Figure 6.**
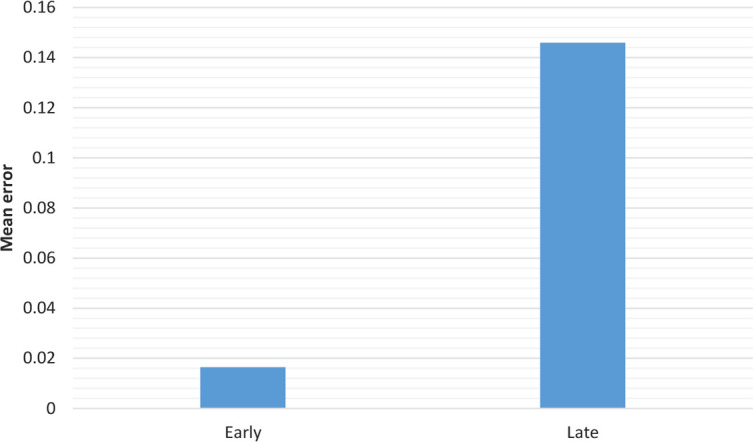
The errors for early and late after 3000 epochs

This simulation was similar to simulation 1, but the numbers of epochs in both groups were twice, and the learning rate was 0.05. As shown in Figure 6, the error of early was much less than that of late and independent of the learning rate. Figure 6. The Errors for Early and Late After 300 Epochs With Learning Rate 0.05

#### 3.2.5. Simulation 5

**Figure 7.**
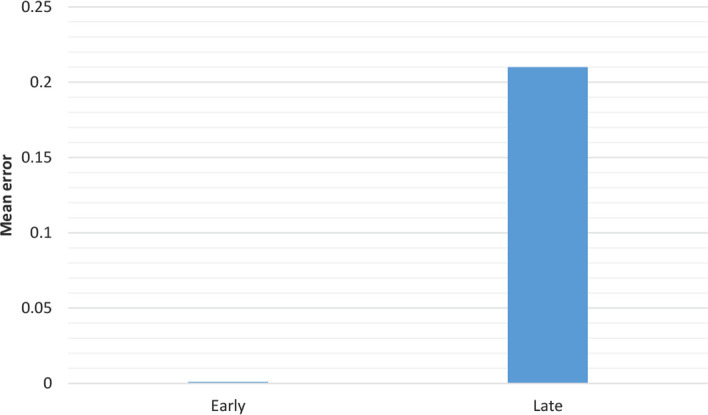
The errors for early and late after 3000 epochs

Here, the aim was to control the momentum, the value that speeds up the learning. Without momentum, after 3000 epochs (1500 early and 1500 mixed), AoA effect was observed similar to the other simulations, as shown in Figure 7.

#### 3.2.6. Simulation 6: Relationship between AoA and brain damage

**Figure 8.**
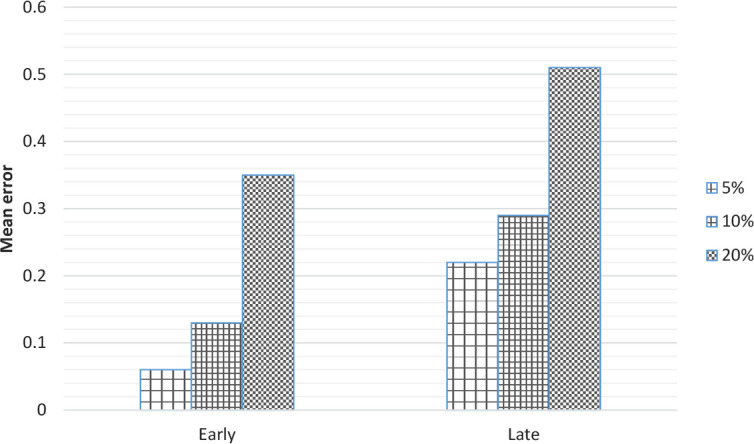
The effect of lesion on AoA at 3 levels of severity (5%, 10%, and 20%)

In this simulation, the relationship between brain damage and AoA (Ellis, & Morrison, 1998) was simulated. The trained model in simulation 1 was lesioned at three levels of severity (5%, 10%, and 20%). Two methods of lesioning were used (both had the same effects): Setting weights to 0, and adding small number of noises to hidden neurons. In each one of the 20 samples, a random portion of neurons in the hidden layer was lesioned at each of the three levels of severity. The obtained result showed that errors raised in the late than the early group. And there was an increase in error, as a function of severity of lesion. Thus, as observed in the diseases such as aphasia and dementia, the effect of damage in the late experience was more than that of an early one (Figure 8).

#### 3.2.7. Simulation 7: The nature of AoA effect

**Figure 9.**
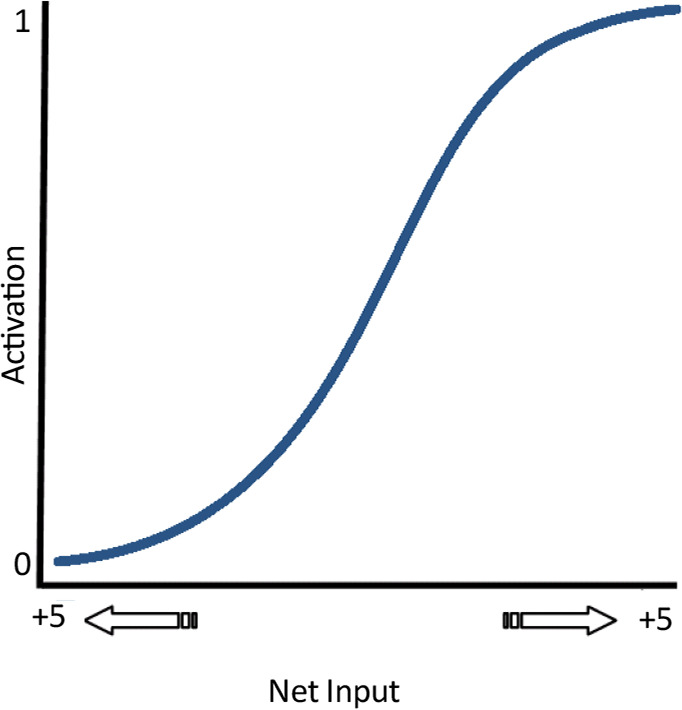
The logistic activation function The slope of the function is greatest, when its input is around 0 as in the start of training, while it decreases in both positive and negative directions as training proceeds.

This is about the nature of AoA effect. As noted earlier, there is more change in the start of learning by error back-propagation due to initial random weights and using sigmoid activation function. Activation is in its highest level when the inputs are around zero (thus at the beginning of learning and in the case of early items) and there is little space to change as learning progresses (Figure 9).

**Figure 10.**
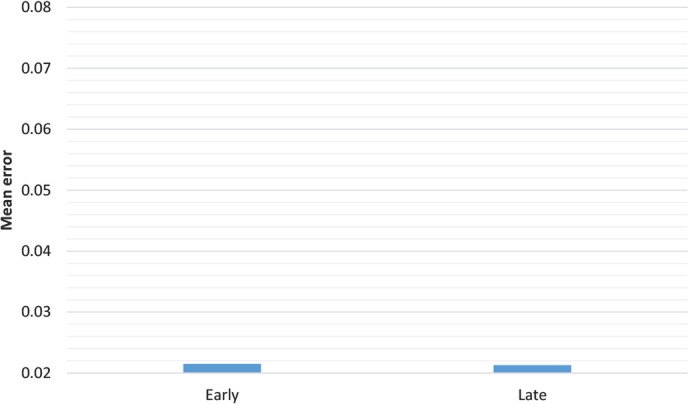
The effect of a modified algorithm, quick prop, on AoA

It was mentioned by Ellis and Rambon-Ralph (2000), but not practically shown. Here, with another algorithm called Quick prop (Fahlman, 1987), the simulation 1 was replicated. Since this algorithm prevents the activation of function from moving to its extreme value, it causes flexibility in the network and eliminates AoA (Figure 10). But Ellis and Rambon-Ralph (2000) showed AoA even using this algorithm and it is, presumably, due to using random patterns instead of real words.

## 4. Discussion

In the first part of the current study, the effect of AoA on word reading and controlling other factors was shown in the 5^th^ grade students. They read words initially learned in the first grades better than the ones learned in the later grades. The consistency covariate was significant and consistent with the assumption of Zevin and Seidenberg (2004). Thus, AoA is mainly due to inconsistent words. Age of acquisition affects the results due to a loss of plasticity related to successful mastering of a task. This phenomenon can occur in several types of learning tasks including reading. It happens due to an interaction between AoA and consistency shown by covariance analysis in the current study.

When a new word is learned after an old one, it can catastrophically affect the learning of the first one if the first one is not presented even for refreshing its learning. But if the first word is presented alongside the second one, it keeps its superiority even after a long time.

As an explanation of AoA by connectionist models, it is noteworthy that in such models, the weights are initially assigned to random values and output units take values of 1 or 0. The weight adjustments that occur during back-propagation with a logistic activation function are proportional to the unit activation (Sohrabi, 2002).Thus, these adjustments mostly occur with the activations in the middle of the logistic function (inputs are around 0), as it happens when small random weights are used for early initialization of the network. Therefore, the plasticity is involved in learning when the early-trained patterns are lost. This effect resembles inflexibility of human brain to new learnings such as infrequent and new words, despite its great flexibility during this age following neurofeedback (Rahmati, Rostami, Zali, Nowicki, & Zare, 2014) and education (Klingberg, 2010; Kranfnick et al., 2011).

When the model was lesioned by removing some hidden units to see its effect on word reading, words learned earlier were less vulnerable to damage compared to the ones learned later. These effects can be explained by considering the nature of learning in neural networks trained by error back-propagation as mentioned above, i e, early learning uses more resources and leaves less for later learning, making it prone to be destroyed by damages.Moreover, there was a failure in replicating previous simulation of AoA in English reading by an algorithm called Quick prop (Fahlman, 1989) for Farsi, presumably due to the flexibility in the network that affects AoA.

This happened in simulating AoA in Farsi, perhaps it is more regular than English. But Ellis and Lambon-Ralph (2000) showed AoA even using this algorithm and it is, presumably, due to using random patterns instead of real words. The problem with using random patterns was also shown by Zevin and Seidenberg (2004). So, the relationship between input and output are arbitrary instead of being quasi-regular as in the case of word naming. This gets even more important knowing that the AoA effect mainly occurs in irregular domains, as in less predictable Chinese characters (Chen, Zhou, Dunlap & Perfetti, 2007), less practiced reading aloud (Zevin & Seidenberg, 2004), and less regular stress in Italian word reading (Wilson et al., 2012).

## Ethical Considerations

### Compliance with ethical guidelines

All procedures performed in studies involving human participants were in accordance with the ethical standards of the institutional and/or national research committee and with the 1964 Helsinki declaration and its later amendments or comparable ethical standards.

## References

[B1] Chen B. G. Zhou H. X. Dunlap S. Perfetti C. A. (2007). Age of acquisition effects in reading Chinese: Evidence in favor of the arbitrary mapping hypothesis. British Journal of Psychology, 98(Pt 3), 499–506. [DOI:10.1348/000712606X165484] [PMID] 17705943

[B2] Carroll J. B. White M. N. (1973). Word frequency and age of acquisition as determiners of picture-naming latency. The Quarterly Journal of Experimental Psychology, 25(1), 85–95. [DOI:10.1080/14640747308400325]

[B3] Coltheart M. Davelaar E. Jonasson K. Besner D. (1977). Access to the internal lexicon. In Dornic S. (Ed.), Attention & Performance VI (pp. 535–55). Hillsdale, NJ: Erlbaum.

[B4] Coltheart V. Laxon V. J. Keating C. (1988). Effects of word imageability and age of acquisition on children’s reading. British Journal of Psychology, 79(1), 1–12. [DOI:10.1111/j.2044-8295.1988.tb02270.x]

[B5] Daneshparvar Z. Daliri M. R. (2012). A simulation based study of dorsal cochlear nucleus pyramidal cell firing patterns. Basic and Clinical Neuroscience, 3(2), 22–31.

[B6] Ellis A. W. Lambon Ralph M. A. (2000). Age of acquisition effects in adult lexical processing reflect loss of plasticity in maturing systems: Insights from connectionist networks. Journal of Experimental Psychology: Learning, Memory and Cognition, 26(3), 1103–23. [DOI:10.1037/0278-7393.26.5.1103] [PMID] 11009247

[B7] Ellis A. Morrison C. (1998). Real age-of-acquisition effects in lexical retrieval. Journal of Experimental Psychology: Learning, Memory and Cognition, 24(4), 515–23. [DOI:10.1037/0278-7393.24.2.515] [PMID] 9530847

[B8] Fahlman S. (1989). Fast learning variations on back-propagation: An empirical study. In Touretzky D. Hinton G. Sejnowski T. (Eds.), Proceedings of the 1988: Connectionist Models (pp. 38–51). San Mateo: Morgan Kaufman Publishers.

[B9] Flege J. E. Yeni-Komshian G. H. Liu S. (1999). Age constraints on second-language acquisition. Journal of Memory and Language, 41(1), 78–104. [DOI:10.1006/jmla.1999.2638]

[B10] Friston K. Frith C. (2015). Active inference, communication and hermeneutics. Cortex, 68(2), 129–43. [DOI:10.1016/j.cortex.2015.03.025] [PMID] [PMCID] 25957007 PMC4502445

[B11] Gerhand S. Barry C. (1998). Word frequency effects in oral reading are not merely age-of-acquisition effects in disguise. Journal of Experimental Psychology: Learning, Memory and Cognition, 24(4), 267–83. [DOI:10.1037/0278-7393.24.2.267]

[B12] Izura C. Pérez M. Agallou E. Wright V. Marín J. Stadthagen González H. (2011). Age/order of acquisition effects and the cumulative learning of foreign words: A word training study. Journal of Memory and Language, 64(1), 32–58. [DOI:10.1016/j.jml.2010.09.002]

[B13] Krafnick A. J. Flowers D. L. Napoliello E. M. Eden G. F. (2011). Gray matter volume changes following reading intervention in dyslexic children. NeuroImage, 57(3), 733–41. [DOI:10.1016/j.neuroimage.2010.10.062] [PMID] [PMCID] 21029785 PMC3073149

[B14] Klingberg T. (2010). Training and plasticity of working memory. Trends in Cognitive Sciences, 14(7), 317–24. [DOI:10.1016/j.tics.2010.05.002] [PMID ] 20630350

[B15] Ludvig E. A. Sutton R. S. Kehoe E. J. (2012). Evaluating the TD model of classical conditioning. Learning and Behaviour, 40(1), 305–19. [DOI:10.3758/s13420-012-0082-6] [PMID] 22927003

[B16] Markson L. Bloom P. (1997). Evidence against a dedicated system for word learning in children. Nature, 385(3), 813–5. [DOI:10.1038/385813a0] [PMID] 9039912

[B17] McCandliss B. D. Posner M. I. Givon T. (1997). Brain plasticity in learning visual words. Cognitive Psychology, 33(4), 88–110. [DOI:10.1006/cogp.1997.0661]

[B18] McClelland J. L. McNaughton B. L. O'reilly R. C. (1995). Why there are complementary learning systems in the hippocampus and neocortex: insights from the successes and failures of connectionist models of learning and memory. Psychological Review, 102(3), 419–57. [DOI:10.1037/0033-295X.102.3.419] [PMID ] 7624455

[B19] Morrison C. M. Ellis A. W. (1995). Roles of word frequency and age of acquisition in word naming and lexical decision. Journal of Experimental Psychology: Learning, Memory and Cognition, 21(1), 116–53. [DOI:10.1037/0278-7393.21.1.116]

[B20] Rahmati N. Rostami R. Zali M. R. Nowicki S. Zare J. (2014). The effectiveness of neurofeedback on enhancing cognitive process involved in entrepreneurship abilities among primary school students in District No. 3 Tehran. Basic and Clinical Neuroscience, 5(4), 277–84. [PMID] [PMCID] 27284392 PMC4656933

[B21] Rumelhart D. E. Todd P. M. (1993). Learning and connectionist representations. In Meyer D. E. Kornblum S. (Eds.), Attention and Performance XIV: Synergies in Experimental Psychology, Artificial Intelligence, and Cognitive Neuroscience (pp. 3–30). Cambridge: MIT Press.

[B22] Rumelhart D. E. Hinton G.E. Williams R. J. (1986). Learning internal representations by error propagation. In Rumelhart D. E. McClelland J. L. PDP Research Group (Eds.), Parallel Distributed Processing: Explorations in the Microstructure of Cognition, Volume 1: Foundations (pp. 318–62). Cambridge: MIT Press.

[B23] Seidenberg M. S. Waters G. S. Barnes M. A. Tanenhaus M. K. (1984). When does irregular spelling or pronunciation influence word recognition. Journal of Verbal Learning and Verbal Behavior, 23(3), 383–404. [DOI:10.1016/S0022-5371(84)90270-6]

[B24] Seidenberg M. S. McClelland J. L. (1989). A distributed, developmental model of word recognition and naming. Psychological Review, 96(4), 523–68. [DOI:10.1037/0033-295X.96.4.523] [PMID] 2798649

[B25] Sohrabi A. (1999). [The effects of frequency and consistency factors on visual word recognition among normal and mental retarded primary school students: A developmental cognitive approach (Persian) [MA. thesis]. Tehran: University of Tehran.

[B26] Sohrabi A. (2001). A distributed developmental connectionist approach to word naming in normal, dyslexic and mental-retarded readers. Paper presented at The 1st International Conference on Cognitive Sciences; 15 June 2001; Tehran, Iran.

[B27] Sohrabi A. (2002). Cognitive deficits in schizophrenia: A connectionist approach. Poster presented at 12th Annual Meeting of Canadian Society for Brain, Behavior and Cognitive Sciences; 30 January 2002; Vancouver, Canada. [PMID]

[B28] Sohrabi A. West R. L. (2009). Positive and negative congruency effects in masked priming: A neuro-computational model based on representation, attention, and conflict. Brain Research, 1289, 124–32. [DOI:10.1016/j.brainres.2009.07.004] [PMID] 19607818

[B29] Soltanzadeh M. J. Daliri M. R. (2014). Evaluation of phase locking and cross correlation methods for estimating the time lag between brain sites: A simulation approach. Basic and Clinical Neuroscience, 5(3), 205–11. [PMID] [PMCID] 25337381 PMC4202548

[B30] Werker J. F. Tees R. C. (1984). Cross-language speech perception: Evidence for perceptual reorganization during the first year of life. Infant Behavior & Development, 7(1), 49–63. [DOI:10.1016/S0163-6383(84)80022-3]

[B31] Wilson M. A. Ellis A. W. Burani C. (2012). Age-of-acquisition affects word naming in Italian only when stress is irregular. Acta Psychologia, 139(3), 417–24. [DOI:10.1016/j.actpsy.2011.12.012] [PMID] 22321454

[B32] Zevin J. Seidenberg M. (2004). Age-of-acquisition effects in reading aloud: Tests of cumulative frequency and frequency trajectory. Memory and Cognition, 32(1), 31–8. [DOI:10.3758/BF03195818] [PMID] 15078042

[B33] Zorzi M. Houghton G. Butterworth B. (1998). Two routes or one in reading aloud? A connectionist dual process model. Journal of Experimental Psychology: Human Perception and Performance, 24(2), 1131–61. [DOI:10.1037/0096-1523.24.4.1131]

